# Identification of Extracellular Segments by Mass Spectrometry Improves Topology Prediction of Transmembrane Proteins

**DOI:** 10.1038/srep42610

**Published:** 2017-02-13

**Authors:** Tamás Langó, Gergely Róna, Éva Hunyadi-Gulyás, Lilla Turiák, Julia Varga, László Dobson, György Várady, László Drahos, Beáta G. Vértessy, Katalin F. Medzihradszky, Gergely Szakács, Gábor E. Tusnády

**Affiliations:** 1 Institute of Enzymology, RCNS, Hungarian Academy of Sciences, Magyar Tudósok krt 2, Budapest, H-1117 Hungary; 2 Department of Applied Biotechnology and Food Sciences, Budapest University of Technology and Economics, Szent Gellért tér 4, Budapest, H-1111, Hungary; 3 Department of Biochemistry and Molecular Pharmacology, Perlmutter NYU Cancer Center, New York University School of Medicine, 522 First Avenue, SRB 1107, New York, NY 10016, USA; 4 Laboratory of Proteomics Research, Biological Research Center of the Hungarian Academy of Sciences, Temesvari krt. 62, Szeged, H-6726, Hungary; 5 Institute of Organic Chemistry, RCNS, Hungarian Academy of Sciences, Magyar Tudósok krt 2, Budapest, H-1117 Hungary

## Abstract

Transmembrane proteins play crucial role in signaling, ion transport, nutrient uptake, as well as in maintaining the dynamic equilibrium between the internal and external environment of cells. Despite their important biological functions and abundance, less than 2% of all determined structures are transmembrane proteins. Given the persisting technical difficulties associated with high resolution structure determination of transmembrane proteins, additional methods, including computational and experimental techniques remain vital in promoting our understanding of their topologies, 3D structures, functions and interactions. Here we report a method for the high-throughput determination of extracellular segments of transmembrane proteins based on the identification of surface labeled and biotin captured peptide fragments by LC/MS/MS. We show that reliable identification of extracellular protein segments increases the accuracy and reliability of existing topology prediction algorithms. Using the experimental topology data as constraints, our improved prediction tool provides accurate and reliable topology models for hundreds of human transmembrane proteins.

Transmembrane proteins (TMPs) are located in the lipid bilayer of the plasma membrane or the organelle membranes. According to the most recent proteome data, 20–30% of the ORFs encode TMPs^1^,^2^,^3^,^4^ containing at least one predicted transmembrane segment (TMS). TMPs are essential in many different biological processes, such as compartmentalization, intracellular communication, vesicle trafficking, ion transport, protein translocation/integration or the propagation of cellular signals. About 55% of the drugs currently approved by the Food and Drug Administration (FDA) target TMPs^5^. In the era of rational drug design, detailed structural information of TMPs would be paramount to the success of drug discovery. Unfortunately, determination of high resolution TMP structures remains an exceedingly difficult task that explains why only 3% of the currently determined structures are TMPs^6^ (considering only structures with no homologous proteins).

In lack of high resolution structures, topology predictions providing information about the position and orientation of transmembrane (TM) regions and the interconnecting loops relative to the membrane remain vital in 3D structure prediction algorithms^7^,^8^. Initially, prediction methods were based on the physicochemical properties of amino acids and the “positive inside” rule^9^. Later, supervised machine learning algorithms have increased the accuracy of the predictions, but since these methods depend on the particular training sets, they usually underperform on test sets containing new protein families^10^. In addition to accuracy, it is important to also consider the reliability of the prediction methods. In this study, accuracy is defined as the percent of proteins with correctly predicted topology in a given set, whereas reliability reflects the probability that a given prediction is correct^3^,^11^. It is well established that the incorporation of experimentally determined topology data results in a significant increase in the accuracy of topology predictions^12^,^13^,^14^. Recently, we have launched the Constrained Consensus TOPology (CCTOP) (http://cctop.enzim.ttk.mta.hu)^11^ prediction algorithm that couples 10 different state-of-the-art prediction methods with experimental information related to homologous proteins listed in the Topology Database of Transmembrane Proteins (TOPDB) database (http://topdb.enzim.hu)^15^. CCTOP also defines a reliability score for each prediction, which was proven to correlate with prediction accuracy. CCTOP was used to create the Human Transmembrane Proteome database (http://htp.enzim.hu), which contains the most accurate topology prediction of human TMPs according to data obtained on a human TMP benchmark set^3^.

Several experimental approaches have been developed for the determination of topologies^15^,^16^. Topology can be mapped by limited proteolysis, and the position of epitopes in native proteins may also be determined using antibodies^17^,^18^,^19^. In both experimental setups, the identification of extracellular segments is based on the inability of proteases or antibodies to penetrate the plasma membrane^20^,^21^. Another strategy relies on the detection of *N*- and *O*-glycosylation sites, targeting natural modifications of specific positions within the extracellular or lumenal parts of proteins^22^,^23^. Several mass spectrometry techniques have been applied for the characterization of glycosylated segments^22^,^23^,^24^,^25^,^26^, and it has also been shown that the glycosylation pattern of TMPs can be exploited to reliably predict the topology for transmembrane proteins^15^,^26^. The topological position of amino acids may also be determined by site directed mutagenesis or by the insertion of various tags including fluorescent proteins^27^,^28^ or reporter enzymes (alkaline phosphatase^29^,^30^,^31^,^32^, β-galactosidase^32^, or β-lactamase^33^). As seen with the native epitope techniques, glycosylation events can be monitored by SDS-PAGE and Western blotting^34^, whereas accessibility of the epitopes (HA^34^,^35^, FLAG^17^, Myc^27^,^36^) may be determined in both intact and permeabilized cells with specific monoclonal antibodies. An obvious caveat of these approaches is that the inserted epitope-tags or glycosylation sites may alter the topology and the function of the studied proteins; moreover, the results are often misinterpreted^10^. The smallest artificial modifications are single amino acid changes such as the insertion or mutation of single cysteine or lysine residues introduced in Cys-less or Lys-less sequence stretches^17^,^36^,^37^. However, the topology of such modified proteins may also become aberrant. While the above experimental approaches may differ in reliability and sensitivity due to the complexity of the procedures, most cannot be used for proteome-wide studies. A compilation of experimental methods used for TMP topology determination is listed in the TOPDB database^15^,^38^, which uses Protein Data Bank (PDB, http://www.rcsb.org)^6^, Protein Data Bank of Transmembrane Proteins (PDBTM, http://pdbtm.enzim.hu)^2^,^39^,^40^ and PubMed as sources of experimentally determined topology data.

In this study, our aim was to expand the available experimental topology data to provide further input for the CCTOP algorithm. In particular, we wanted to design an experimental method that allows the high throughput identification of extracellular lysine side chains based on their modification with a membrane-impermeable labeling agent. We show that partial labeling of TMPs generates sufficient constraints to significantly increase the reliability and accuracy of topology predictions. To the best of our knowledge, this is the first attempt to combine experimental and computational approaches to produce topology models for hundreds of human TMPs.

## Results

### Labeling and enrichment of extracellular protein segments

**Figure 1:**
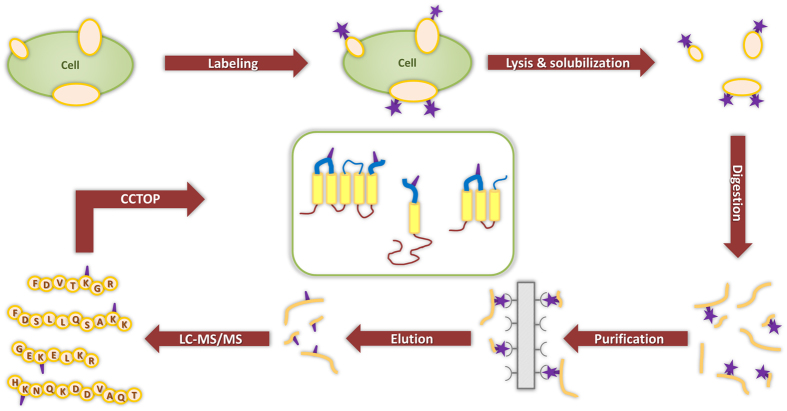
Flowchart of the method developed to identify extracellular lysine residues. Isolated cells were labeled with a membrane-impermeable, lysine specific labeling agent (sulfo-NHS-SS-biotin). The membrane fraction was purified, solubilized and digested with different proteolytic enzymes. The modified peptides were isolated on a neutravidin agarose resin, then eluted and sequenced by tandem mass spectrometry. Labeled positions were used as extracellular constraints in the CCTOP topology prediction algorithm.

We have optimized a well-established labeling method^41^,^42^,^43^,^44^,^45^,^46^,^47^ to enhance topology prediction of hundreds of endogenous proteins by high throughput experimental topology data (Fig. 1). This method relies on the selective chemical tagging of cell surface proteins by Sulfosuccinimidyl-2-(biotinamido)ethyl-1,3-dithiopropionate (sulfo-NHS-SS-biotin), which is a membrane impermeable reagent labeling only extracellular amino termini and lysine side chains. While covalent surface labeling has been used to identify cell surface proteins^42^,^43^,^44^,^45^,^46^ and to determine topology of particular protein (e.g. IFITM1^41^), here our aim was to label extracellular segments of the majority of surface exposed transmembrane proteins to improve topology prediction of the transmembrane proteome. Labeling conditions were optimized to minimize false positives, i.e. labeling of intracellular segments of TMPs. Cell-surface biotinylation of Chinese hamster ovary (CHO) cells was verified by confocal microscopy, which showed homogenous fluorescent labeling of the cell surface and no signal in the cytoplasm (Supplementary Figures 1 and 2). Importantly, treatment with sulfo-NHS-SS-biotin did not compromise the integrity of the cells, as suggested by measuring cell death by propidium iodid uptake (Supplementary Figure 3A). Labeling of intact cells was followed by the solubilization and digestion of the membrane preparations and the affinity enrichment of the modified peptides. Samples corresponding to different stages of the purification process were blotted onto a PVDF membrane to allow semi-quantification of biotinylated peptides. Results presented in Supplementary Figure 4 show that the binding capacity of the neutravidin beads did not limit the enrichment of the biotinylated peptides. Biotin contents of samples taken before affinity isolation (Supplementary Figure 4 C1,3,5) and samples from fractions after affinity binding (Supplementary Figure 4 C2,4,6) clearly show that the biotinylated components remained bound to the neutravidin beads.

Peptides were eluted from the neutravidin agarose affinity columns using reducing agents or formic acid in the absence or presence of alkylating agents, which were added to block peptide aggregation through disulphide bridges. The method results in the covalent labeling of extracellular primer amines by a thioacyl (+87.998 Da) or the thioacyl-Biotin (+389.090 Da) or carbamidomethylthio-propanoyl group (+145.020 Da) or Thio(AE) (+131.040 Da) or Thio(NEM) (+213.045 Da) (Supplementary Table 1 and 2).

### Identification of the labeled peptides by tandem mass spectrometry

Using the optimized protocol, we labeled human red blood cells (RBC), HL60 and K562 cells. The list of peptides carrying the modified lysines is shown in Supplementary Table 2, and the supporting spectra can be viewed on the Protein Prospector public website (prospector.ucsf.edu), search keys are listed in Supplementary Table 2/*Online Repository Links* sheet.

**Figure 2:**
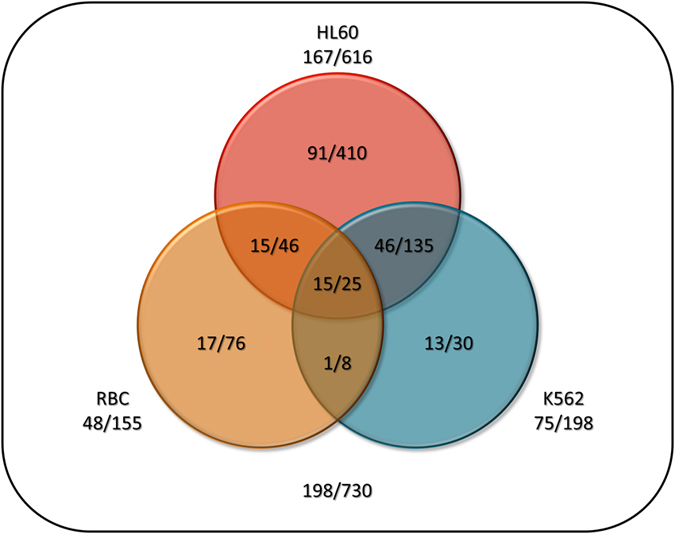
Summary of the labeled TM proteins/positions in different cell lines. Venn diagram showing the number of individually labeled TMPs in the three cell lines used in the study.

Altogether 17493 peptides containing covalently modified lysines were detected by liquid chromatography tandem-mass spectrometry (LC/MS/MS, Supplementary Table 2). Modified peptides from the various searches were mapped to unique proteins, and the modifications in unique proteins were counted. We considered only those lysines that were identified in at least three independent labeling experiments. Using this filter, we identify 730 (47%) positions in 198 (38%) TMPs, 593 (38%) positions in 212 (40%) intracellular and 250 (16%) positions in 114 (22%) extracellular proteins. Regarding TMPs only, 167 TMPs with 616 labeled positions, 75 TMPs with 198 labeled positions and 48 TMPs with 155 labeled positions were detected from HL60, K562 cell lines and RBC, respectively (Fig. 2 and Supplementary Table 3). Regarding all the homologous TMPs that can produce the labeled peptides, our experiments provided topology data for 2776 human TMPs in the UniProt database (Supplementary Table 3).

### Validation of the experimental results

To validate our results, we compared the topological location of the labeled lysine residues to independent experimental results reported in the literature^15^. While no prior experimental topology data was available for 85 proteins, we identified 450 out of the 730 labeled lysines in 113 proteins whose topology could be confirmed by independent experiments. Of these, 98.7% of the positions were correctly classified. It is important to note that the 113 proteins whose topology could be confirmed are not sequentially similar or identical, thus this validation is not biased by selecting only one specific type of TMPs from the TOPDB database.

### Characterization of the labeled lysine residues

To gain insight into the efficiency of the labeling method, we compared the sequential environment of the labeled lysines with that of the extracellular lysines that were expected to be labeled but were not detected in our experiments. The sequence logos of the surrounding 20 amino acids clearly show that our method preferentially identified those lysines that are followed by positively charged amino acids that serve as tryptic cleavage sites and ensure the appropriate peptide size for MS analysis (Supplementary Figure 5). Moreover, the highest value (the smallest entropy) at position −1 of the labeled peptides suggests that the proximal amino acid may alter the chemical reaction of the labeling process. We also calculated the length distributions of those extracellular segments that were identified by covalently modified lysines and of those extracellular segments that would be identified if the same number of lysines have been modified randomly (Supplementary Figure 6). The two distributions are not significantly different, suggesting that reactivity with sulfo-NHS-SS-biotin is not influenced by the length of the extracellular loops or domains.

### Validation of the overall method

**Figure 3:**
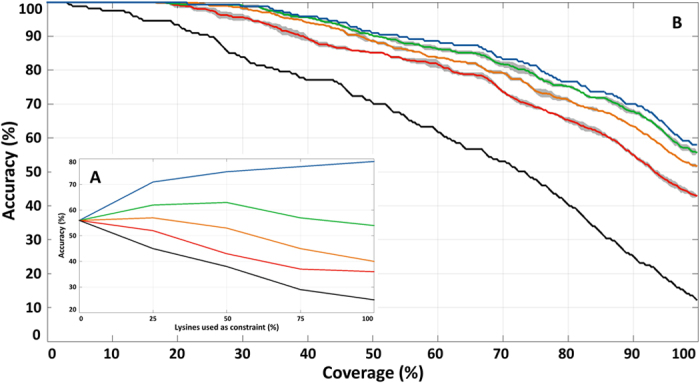
Effect of lysine constraints on the accuracy of topology prediction. Effect of constraints on the topology prediction accuracy of CCTOP on the experimental benchmark set. (**A**) Prediction accuracy versus percent of extracellular lysines used as constraints in the prediction. 0, 4, 8, 12, 16% of the used extracellular lysines were replaced by intracellular lysines (blue, green, orange, red and black line, respectively) (**B**). Predictions were sorted according to their reliability values, then the accuracies were calculated on proteins with the largest 1, 2, 3 … 333 reliability values, represented as coverage from 0 to 100% on the x-axis of the plot. The colors of the curves are coded according to the ratio of randomly selected extracellular lysine (blue, green, orange, red and black for 100, 75, 50, 25 and 0%, respectively). Averages are plotted with continuous lines, standard deviations are shaded.

To measure the potential impact of our experiments on topology predictions, we analyzed a human TMP benchmark set consisting of 333 human TMP sequences^3^. The topology of these TMPs was established based on the available 3D structures of the same or homologous proteins. The set contains 8099 extracellular and 4892 intracellular lysines. Using the CCTOP algorithm, we simulated topology predictions taking into account an increasing number of extracellular lysines as constraints^11^. To avoid any bias, all further computational and experimental constraints were neglected, and only the selected lysines were considered. We selected 25%, 50% and 75% of extracellular lysines by chance, and compared the results of the predictions to the established topology of the benchmark TMPs, using only these randomly selected extracellular lysine residues as constraints (Fig. 3A, blue line). The randomizations were repeated 50 times to calculate the average and the standard deviation of the prediction accuracy and reliability (Fig. 3B, Supplementary Figure 7). To assess the theoretical limits of our approach, a simulation was run in which all extracellular lysines were considered as constraints (100% on the plots). As expected, the accuracy of the topology predictions was significantly improved by involving extracellular lysines as constraints (Fig. 3). The simulations suggest that the maximal benefit is a 23% increase in the prediction accuracy (from 56% to 79%) (Fig. 3A, blue line), which would occur with the labeling of all extracellular lysines. By limiting the constraints to 20% of the extracellular lysines (corresponding to the percent of labeled lysines in our experiments), the accuracy of the topology predictions is still increased by 14% (from 56% to 70%). To simulate the effect of erroneously identified positions on prediction accuracy, we corrupted the prediction algorithm by replacing 4, 8, 12 and 16% of the randomly selected 25, 50, 75 and 100% extracellular lysines with intracellular lysines. As shown in Fig. 3A, false positive constrains have a drastic effect, resulting in an actual decline of the prediction accuracy.

## Discussion

Sulfo-NHS-SS-biotin has been extensively used to determine the orientation of unique protein termini^41^ or to identify cell surface proteins in different cell lines, such as blood and lymphatic vascular endothelial cells^42^, mesenchymal stromal cells^43^, hepatoma cells^44^, B-cell precursor acute lymphoblastic leukemia^45^, melanoma cells^46^ and pancreatic cancer cells^47^. Here we used this well-established labeling agent for the identification of extracellular lysine residues of transmembrane proteins in order to increase the topology prediction accuracy and the reliability of the CCTOP algorithm. We assessed the potential of the experimental approach by modelling the impact of constraints on the accuracy and reliability of the CCTOP predictions using a TMP benchmark set. As expected, both values increased by applying additional constraints, although not in a linear fashion (Fig. 3 and Supplementary Figure 7). Near half of the maximal increase was achieved by using only 20% of the potential extracellular lysines as constraints. Moreover, the simulations revealed that the prediction accuracy can be increased only if experimental data are free or almost free of error. Unfortunately, labeling of intracellular lysines of TMPs results in a significant deterioration of the prediction accuracy limiting experimental strategies that would increase false positive hits. In view of the simulations, we optimized the labeling procedure to minimize the risk of labeling intracellular residues of TMPs. In particular, we used a membrane-impermeable reagent and optimized the experimental conditions to ensure maximal extracellular biotinylation without intracellular labeling of TMPs (Supplementary Figures 1–3). We also verified that the binding capacity of neutravidin agarose columns was not exhausted and the bound biotinylated peptides are fully removed for MS analysis (Supplementary Figure 4). Importantly, we purified labeled peptides rather than labeled proteins and did not consider unlabeled peptides as hits. This strategy lowered the number of identified proteins, but ensured a low false discovery rate.

To validate our results, we collected published information on the exact localizations of the labeled segments. 450 out of the 730 labeled lysines could be compared to independent experimental data – of which 98.7% was confirmed to be located extracellularly. Based on this result, we are confident that our experimental data may be used as constraints in the CCTOP algorithm to enhance the large scale topology prediction of human TMPs.

In our experiments we identified 730 topological positions for 198 TMPs in three cell lines. Not all of the known TMPs were identified from these cell lines possibly because of the following reasons: i) lack or inaccessibility of primary amines in the extracellular domain of membrane spanning proteins; ii) too short or too long peptides were produced during the digestion, preventing identification by LC/MS/MS; iii) post-translational modifications may prevent the identification of peptides and proteins; iv) different abundance of proteins in a particular sample (dynamic range problem)^42^.

Besides the extracellular part of the TMPs, some abundant intracellular and extracellular proteins were also labeled (Supplementary Table 2). It is important to note that the observed intracellular labeling was restricted to intracellular proteins, and did not affect intracellular segments of TMPs. Labeled cytosolic proteins are abundant and likely originate from damaged cells, that are attached to the cell surface in normal blood circulation^48^. For example, we detected labeled histone proteins that are bound to neutrophil extracellular traps containing DNA, histones and cell-specific granule proteins^49^. Other labeled cytosolic proteins have also been reported as adsorbed proteins of the particular cell surface (tubulin, actin), which were also present as contaminants in our data^50^,^51^. A similar level of intracellular protein labeling was reported by Hofmann *et al*. who used Cell Surface Capture (CSC) analysis to study surface proteins from four Hodgkin and four non-Hodgkin lymphoma cell lines^52^. Since the labeling of intracellular lysines was restricted to intracellular proteins that were labeled outside of the cells, we were confident that the experimental strategy was in keeping with the expected low false positive hit rate.

Our experimental data yielded 6 conflicting positions, 3 of which belong to two proteins that have homologous protein structures in the PDBTM database. For the topology assessment of ADT2_HUMAN (ADP/ATP translocase 2) protein we used a closely related structure, 2C3E (89% sequence identity)^53^, which suggested that Lys-147 is cytosolic and Lys-23 is localized in the membrane. Lys-23, which is detected 41 times in our experiments, is located in the cavity of the outward open structure, which is likely accessible to the Sulfo-NHS-SS-biotin reagent. Similarly, labeling of Lys-147 (detected 6 times) can be also explained by the penetration of the biotinylation agent through the open gate (Supplementary Figure 8).

Two positions with conflicting data were identified in the GTR1_HUMAN (Solute carrier family 2, facilitated glucose transporter member 1) protein, which were analyzed in the PDB:4PYP structure (99% sequence identity)^54^. This structure captures the protein in an inward open conformation where the coordinates of the bound ligand were also available. Positions Lys-245 and Lys-256 (both detected 3 times) belong to an α-helix positioned in front of the cytosolic entrance of the gate, suggesting that the labeling agent could have reached these lysines from the extracellular compartment in the open conformation. Interestingly, on the extracellular side, only Lys-117 was detected more than 50 times. The low level labeling of Lys-38, Lys-183 and Lys-300 is also consistent with the continuous transition between the open and closed states when the moving ligand covers these lysines while passing through the channel.

Cell Surface Capturing by chemical tagging of N-linked glycopeptides has been recently used for the characterization of the cell surface proteome of several cell lines^26^. Since glycosylation occurs only on extra-cytoplasmic segments, glycan-specific purification of pepti.des offers a highly specific method for the identification of extracellularly localized peptides. However, the frequency of extracellular lysines is four times larger than that of the N-X-S/T motifs where the glycolysations happen. Therefore, chemical labeling and identification of extracellular lysines may offer more input for topological predictions. Unfortunately, Lys-cell surface capture technology (Lys-CSC), reported by Hofmann *et al*. showed a very high level labeling of intracellular TMP segments: 17% of lysines can be found on intracellular part and only 83% of the labeled lysines were on the extracellular segments of TMPs (calculated using the topology prediction results of the CCTOP method on the observed TMPs and the unambiguously tagged lysine positions reported in the Supporting materials of Hofmann *et al*.^52^). The parameter optimization, reported in our work, diminished false positive lysine labeling and increased the labeling accuracy to 98.7%, which allowed a significant improvement of the constrained topology prediction.

The topology information gained from our experimental results contributes to a more accurate topology prediction of the human transmembrane proteome. In future studies, we plan to increase the scope of the predictions by including further cell lines and cellular organelles expressing different unique TMPs^26^, combined with different proteases to increase sequence coverage. Further experimental data will provide a better understanding of the topology structure of individual TMPs and will help us to elucidate the structure-function relationship of transmembrane proteins.

## Methods

### Experimental Design and Statistical Rationale

Proteomic study was performed on two human cell lines (HL60 is an acute promyelocytic leukemia cell line, K562 is a chronic myelogenous leukemia cell), and on human red blood cells (RBC). Labeling reactions and downstream purifications were carried out in at least three biological replicates for each sample type. Mass spectrometry measurements were repeated at least three times from each isolation.

### Human samples

The study was approved by the regional ethical committees (Department of Health, Office of Hungarian Government, Budapest, Hungary), and all procedures were performed in accordance with the Declaration of Helsinki. The blood samples were collected after obtaining written consent; sampling was performed at the Hungarian National Blood Transfusion Service^55^.

### Cell Cultures

HL60, K562 and CHO (Chinese hamster ovary) cells were obtained from American Type Culture Collection. CHO cells were cultured in F12 HAM (Sigma, St. Louis, MO, USA) while HL60 and K562 cells were cultured in RPMI (Sigma) supplemented with 50 μg/ml Penicillin-Streptomycin (Gibco, Life Technologies, Carlsbad, CA, USA) and 10% fetal bovine serum (FBS) (Gibco) in a humidified 37 °C incubator with 5% CO_2_ atmosphere.

### Cell isolation

RBC (1–2 × 10^10^ RBC/sample) was isolated from 1–2 ml blood by centrifugation at 500 × g for 5 minutes at 4 °C; pelleted cells were washed with PBS three times to remove contaminating platelets and white-blood cells. HL60 and K562 cells were collected similarly by centrifugation at 500 × g for 5 minutes at 4 °C. In the last washing step, 4 mM iodoacetamide alkylation agent was used for the blocking of free sulfhydryl groups to avoid the production of “piggy-back” disulphide peptides.

### Cell surface labeling

Cell surface biotinylation was performed using 2 mM Sulfo-NHS-SS-biotin (Thermo Fisher Scientific, Waltham, MA, USA) in PBS (pH = 7.4) at 4 °C for 20 minutes. For MS analysis 10^10^ RBCs or 10^8^ HL60 and K562 cells were used for each experiment. The concentration of the labeling agent was optimized by flow cytometry and the labeling was verified by confocal microscopy (Supplementary Methods and Supplementary Figures 1–3). Labeling reaction was stopped with 100 mM Tris(hydroxymethyl) aminomethane hydrochloride (TRIS.HCl) pH = 7.4, 150 mM NaCl for 10 minutes at 4 °C.

### Membrane preparation

RBC membranes (ghosts) were prepared according to the method described by Schatzmann, Rossi and Wolf^55^,^56^,^57^. HL60 or K562 cells were lysed in a hypotonic lysis buffer (10 mM TRIS.HCl pH = 7.5, 0.5 mM MgCl_2_) or Wolf-Schatzmann haemolysis buffer (20 mM TRIS.HCl pH = 7.4, 10 mM KCl, 20 mM sucrose) in the presence of 10 mM iodoacetamide for 10 minutes at 4 °C. The samples were passed several times through a 26-gauge needle. Cell debris and the nuclei were pelleted by centrifugation at 1700 × g for 10 minutes at 4 °C. Membrane fraction was collected by centrifugation of the supernatant at 100000 × g for 1 hour at 4 °C. Pellets were washed twice with Wolf-Schatzmann washing buffer (2 mM TRIS.HCl pH = 7.7, 1 mM KCl, 2 mM sucrose) and resuspended in 100 mM NH_4_HCO_3_ buffer (pH = 8.0). Protein concentration of the membrane preparations was determined by the method of Lowry *et al*.^58^ using bovine serum albumin as a standard.

### Membrane protein solubilization and digestion

Membrane proteins were solubilized in 100 mM NH_4_HCO_3_ buffer (pH = 8.0) containing 0.05–0.1% (w/v) Rapigest surfactant, 10% acetonitrile, 1 mM iodoacetamide and 1 mM 2,2′-thiodiethanol. The latter was used to prevent overalkylation during the overnight digestion. Solubilization was assisted by brief pulses of sonication followed by incubation on ice for 30 minutes. The suspension was incubated with 500 units of PNGaseF (New England Biolabs) for 2 hours at 37 °C before adding trypsin, chymotrypsin or thermolysin in a 1:50 (w/w) protease:protein ratio (the various enzymes were applied on separated samples). The samples were incubated at optimum temperature of the given enzyme (trypsin: 37 °C, chymotrypsin: 30 °C, thermolysin: 70 °C) for 16 hours. Thermolysin and chymotrypsin digestion mixtures were supplemented with 0.5 mM CaCl_2_ or 10 mM CaCl_2_ and 0.5 mM MgCl_2_, respectively. Digestion was stopped by heat inactivation (95 °C for 10 min) followed by the addition of the appropriate enzyme inhibitors to the reaction mixture: 100 μM TLCK, 100 μM TPCK and 10 mM EDTA, 10 mM 1,10-phenanthroline in case of trypsin, chymotrypsin and thermolysin, respectively. Protein samples (10 μg) were loaded on a 12% SDS-PAGE to compare digestion efficiencies. Gels were stained with Coomassie Brilliant Blue.

### Biotinylated peptide isolation

The biotinylated peptides were precipitated on neutravidin agarose beads (Pierce). In order to bind all biotinylated products, saturation of the neutravidin column was monitored by dot-blots (Supplementary Methods and Supplementary Figure 4). Digestion mixtures were incubated with 300–500 μl of packed, equilibrated neutravidin agarose beads for 2 hours at room temperature. Columns were washed extensively to reduce the number of non-specific peptides or contaminants. Washing steps were performed by the following buffers with 5–10 ml (20 bead volumes): 100 mM NH_4_HCO_3_ (pH = 8.0), 5 M NaCl in PBS, 100 mM NH_4_HCO_3_ (pH = 8.0), 100 mM NaHCO_3_ (pH = 11.0) and a final wash with 100 mM NH_4_HCO_3_ (pH = 8.0) at 65 °C. Beads were transferred into a new, equilibrated spin column before the final washing step carried out with 100 mM NH_4_HCO_3_ (pH = 8.0). Enriched peptides were eluted by incubating the beads with 100 mM NH_4_HCO_3_ (pH = 8.0) buffer containing 50 mM DTT or TCEP for 1 hour at room temperature or by incubating the beads with 100 μl of concentrated formic acid (98%) for 1 hour at 37 °C. In order to avoid further disulphide-bridge formation, free sulfhydryls were alkylated either with iodoacetamide (Sigma), N-ethylmaleimide (Sigma), or 2-bromoethylamine (Sigma).

### Mass spectrometry analysis and peptide identification

Peptide mixtures were analyzed by LC/MS/MS using two different instrument setups. In one setting a nanoAcquity (Waters, Milford, MA, USA) Ultrahigh Pressure Liquid Chromatography (UPLC) system was coupled online to an Linear Trap Quadrupole (LTQ)-Orbitrap Elite (Thermo Scientific, Waltham, MA, USA) mass spectrometer. 5 μl (~1/50–1/80) of the peptide mixture was injected onto a Symmetry C18 nanoAcquity UPLC trap column (Waters, 0.18 × 20 mm, 5 μ, 100 Å) with a flow rate of 10 μl/min for 2 min and separated on a BEH300C18 nanoAcquity UPLC column (Waters, 0.075 × 250 mm, 1.7 μm, 300 Å) using a linear gradient of 3–40% of solvent B in 40 or 100 min. Solvent A was 0.1% formic acid in water, solvent B was acetonitrile containing 5% DMSO (dimethyl sulfoxide) and 0.1% formic acid, the flow rate was 300 nl/min). Survey scans measured in the Orbitrap (resolution = 60000) were followed by CID acquisitions in the linear trap, or HCD acquisitions, from the 10 or 5 most abundant multiply charged ions, respectively (normalized collision energy was 35% and dynamic exclusion was enabled for 30 sec exclusion duration). In some cases, the peptide mixture eluted from the Neutravidine gel was further purified or prefractionated using a C18 ZipTip (Millipore).

In another setting we used a Bruker Maxis II ETD Q-TOF (Bremen, Germany) mass spectrometer with CaptiveSpray nanoBooster ionization source coupled to a Dionex Ultimate 3000 NanoLC System (Sunnyvale, CA, USA). Peptides were trapped on Acclaim™ PepMap100™ C18 Nano-Trap column (5 μm, 100 Å, 100 μm × 20 mm, Thermo Fisher Scientific, Waltham, MA, USA) and separated online using a 15 cm Waters Peptide BEH C18 nanoACQUITY 1.7 μm particle size UPLC column and gradient elution (2.5–25% eluent B in 80 min, then 25–45% eluent B in 20 min). Solvent A was water +0.1% formic acid (FA), while solvent B was acetonitrile +0.1% FA. For the MS measurements a fix cycle time of 2.5 sec was used, MS spectra were acquired at 3 Hz in the 150–2200 *m/z* mass range, while CID was performed at 16 Hz for abundant precursors and at 4 Hz for ones of low abundance.

For the LTQ-Orbitrap Elite data, Proteome Discoverer (Thermo, v1.4) or PAVA script^59^ was used for MS/MS peak list generation and database search was executed using ProteinProspector v5.14.1. At least two consecutive searches were performed. First, the complete SwissProt (SwissProt.06.10.2014 (545388/545388 entries searched) protein database was used to identify proteins present in the sample. For the second search the following database was compiled and concatenated with its randomized sequences: the human UniProt (UniProtKB.2015.4.16 (145723/47262724 entries searched) database was concatenated with the SwissProt hits. The data were searched for tryptic (if chymotrypsin or thermolysine were used for digestion the appropriate enzyme was set) peptides with one or two non-specific and 2–3 missed cleavage sites. No constant modification was used but several variable modifications were set: carbamidomethyl (C), oxidation (M), deamidation (NQ), thioacylation (K) and carbamidomethylthio-propanoylation (K). When the peptides were eluted from the beads with formic acid, the thioacyl-Biotin (+389.090 Da) modification of lysine residues was set. When other alkylation than iodoacetamide was performed on separated samples after the DTT or TCEP elution, additional variable modifications were listed for lysine residues: Thio(AE) (+131.04 Da) or Thio(NEM) (+213.045 Da) and on the cysteine residues: aminoethyl (+43.04 Da) or N-ethylmaleimide (+125.048 Da) (Supplementary Table 1). Maximum 3 modifications were permitted per peptides. Mass tolerance was set to 10 ppm for the precursor ions. Fragment ion mass accuracy was set to 0.6 Da for ion trap CID data and 25 ppm for HCD data. The ProteinProspector default acceptance criteria (min. 15 and 22 score and 0.05 and 0.01 max. E value for peptides and proteins, respectively) were used for the evaluation process with manual inspection for labeled Lys containing peptides. The false discovery rate was calculated by dividing the double of the number of the decoy peptide hits with the number of the identified spectra and it was found to be less than 1% in every search result.

For the QTOF data, raw data were first recalibrated using Bruker Compass DataAnalysis software 4.3 (Bruker Daltonik GmbH, Bremen, Germany) for the internal calibrant. MS/MS peak list generation was performed using ProteinScape software 3.1 (Bruker Daltonik GmbH, Bremen, Germany). As above, the samples were first matched with the human SwissProt database (SwissProt 2014_08, 546238/546238 entries searched). Decoy database was generated by Mascot. The parameters of the Mascot search engine were set as follows: semiTrypsin enzyme, maximum 4 missed cleavages, carbamidomethyl (C) as fixed modification and several variable modifications: oxidation (M), deamidation (NQ), thioacylation (K and protein N-term) and carbamidomethylthio-propanoylation (K and protein N-term). MS tolerance was set to 7 ppm; MS/MS tolerance was 0.05 Da. Mascot ion score corresponding to p < 0.05 was 13, however to ensure confident modified peptide identifications, matches were accepted above a score of 24. The false discovery rate was less than 1% in every search result.

### Processing of the MS results

Mass spectrometry experiments yielded confidently identified modified peptides belonging to different proteins (Supplementary Table 2). Since different search engines and databases were used for the data evaluation, we unified the results by mapping all the resulted peptides to UniProt human sequences and filtering to 95% sequence identity of the mapped proteins. We used the CCTOP algorithm to decide if the mapped proteins were indeed TMPs (containing at least one predicted TMS) or not. Positions corresponding to labeled peptides from at least three different biological replicates were considered further (Supplementary Table 3).

## Additional Information

**How to cite this article**: Langó, T. *et al*. Identification of Extracellular Segments by Mass Spectrometry Improves Topology Prediction of Transmembrane Proteins. *Sci. Rep.* **7**, 42610; doi: 10.1038/srep42610 (2017).

**Publisher's note:** Springer Nature remains neutral with regard to jurisdictional claims in published maps and institutional affiliations.

## Supplementary Material

Supplementary Data

Supplementary Table 1

Supplementary Table 2

Supplementary Table 3

## References

[b1] Fagerberg L., Jonasson K., von Heijne G., Uhlén M. & Berglund L. Prediction of the human membrane proteome. Proteomics 10, 1141–1149 (2010). 20175080 10.1002/pmic.200900258

[b2] Kozma D., Simon I. & Tusnády G. E. PDBTM: Protein Data Bank of transmembrane proteins after 8 years. Nucleic Acids Res. 41, D524–529 (2013). 23203988 10.1093/nar/gks1169 PMC3531219

[b3] Dobson L., Reményi I. & Tusnády G. E. The human transmembrane proteome. Biol. Direct 10, 31 (2015). 26018427 10.1186/s13062-015-0061-x PMC4445273

[b4] Wallin E. & von Heijne G. Genome-wide analysis of integral membrane proteins from eubacterial, archaean, and eukaryotic organisms. Protein Sci. 7, 1029–38 (1998). 9568909 10.1002/pro.5560070420 PMC2143985

[b5] Uhlén M. . Proteomics. Tissue-based map of the human proteome. Science 347, 1260419 (2015). 25613900 10.1126/science.1260419

[b6] Sussman J. L. . Protein Data Bank (PDB): database of three-dimensional structural information of biological macromolecules. Acta Crystallogr. D. Biol. Crystallogr. 54, 1078–84 (1998). 10089483 10.1107/s0907444998009378

[b7] Kozma D. & Tusnády G. E. TMFoldWeb: a web server for predicting transmembrane protein fold class. Biol. Direct 10, 54 (2015). 26381605 10.1186/s13062-015-0082-5 PMC4574079

[b8] Kozma D. & Tusnády G. E. TMFoldRec: a statistical potential-based transmembrane protein fold recognition tool. BMC Bioinformatics 16, 201 (2015). 26123059 10.1186/s12859-015-0638-5 PMC4486421

[b9] Heijne G. The distribution of positively charged residues in bacterial inner membrane proteins correlates with the trans-membrane topology. EMBO J. 5, 3021–7 (1986). 16453726 10.1002/j.1460-2075.1986.tb04601.x PMC1167256

[b10] Tusnády G. E. & Simon I. Topology prediction of helical transmembrane proteins: how far have we reached? Curr. Protein Pept. Sci. 11, 550–561 (2010). 20887261 10.2174/138920310794109184

[b11] Dobson L., Reményi I. & Tusnády G. E. CCTOP: a Consensus Constrained TOPology prediction web server. Nucleic Acids Res. 43, W408–12 (2015). 25943549 10.1093/nar/gkv451 PMC4489262

[b12] Tusnády G. E. & Simon I. The HMMTOP transmembrane topology prediction server. Bioinformatics 17, 849–50 (2001). 11590105 10.1093/bioinformatics/17.9.849

[b13] Rapp M. . Experimentally based topology models for E. coli inner membrane proteins. Protein Sci. 13, 937–45 (2004). 15044727 10.1110/ps.03553804 PMC2280059

[b14] Melén K., Krogh A. & von Heijne G. Reliability measures for membrane protein topology prediction algorithms. J. Mol. Biol. 327, 735–44 (2003). 12634065 10.1016/s0022-2836(03)00182-7

[b15] Dobson L., Langó T., Reményi I. & Tusnády G. E. Expediting topology data gathering for the TOPDB database. Nucleic Acids Res. 43, D283–9 (2015). 25392424 10.1093/nar/gku1119 PMC4383934

[b16] van Geest M. & Lolkema J. S. Membrane topology and insertion of membrane proteins: search for topogenic signals. Microbiol. Mol. Biol. Rev. 64, 13–33 (2000). 10704472 10.1128/mmbr.64.1.13-33.2000 PMC98984

[b17] Bai X.-Y. . Membrane topology structure of human high-affinity, sodium-dependent dicarboxylate transporter. FASEB J. 21, 2409–17 (2007). 17426067 10.1096/fj.06-7652com

[b18] Covitz K. M., Amidon G. L. & Sadée W. Membrane topology of the human dipeptide transporter, hPEPT1, determined by epitope insertions. Biochemistry 37, 15214–21 (1998). 9790685 10.1021/bi981128k

[b19] Geyer J. . Cloning and functional characterization of human sodium-dependent organic anion transporter (SLC10A6). J. Biol. Chem. 282, 19728–41 (2007). 17491011 10.1074/jbc.M702663200

[b20] Bakos É. . Membrane topology and glycosylation of the human multidrug resistance-associated protein. J. Biol. Chem. 271, 12322–6 (1996). 8647833 10.1074/jbc.271.21.12322

[b21] Shields D. J., Lehner R., Agellon L. B. & Vance D. E. Membrane topography of human phosphatidylethanolamine N-methyltransferase. J. Biol. Chem. 278, 2956–62 (2003). 12431977 10.1074/jbc.M210904200

[b22] Steentoft C. . Precision mapping of the human O-GalNAc glycoproteome through SimpleCell technology. EMBO J. 32, 1478–1488 (2013). 23584533 10.1038/emboj.2013.79 PMC3655468

[b23] Wollscheid B. . Mass-spectrometric identification and relative quantification of N-linked cell surface glycoproteins. Nat. Biotechnol. 27, 378–86 (2009). 19349973 10.1038/nbt.1532 PMC2829300

[b24] Zielinska D. F., Gnad F., Wiśniewski J. R. & Mann M. Precision mapping of an *in vivo* N-glycoproteome reveals rigid topological and sequence constraints. Cell 141, 897–907 (2010). 20510933 10.1016/j.cell.2010.04.012

[b25] Trinidad J. C., Schoepfer R., Burlingame A. L. & Medzihradszky K. F. N- and O-glycosylation in the murine synaptosome. Mol. Cell. Proteomics 12, 3474–88 (2013). 23816992 10.1074/mcp.M113.030007 PMC3861701

[b26] Bausch-Fluck D. . A mass spectrometric-derived cell surface protein atlas. PLoS One 10, e0121314 (2015). 25894527 10.1371/journal.pone.0121314 PMC4404347

[b27] Wang J. . Membrane topology of human NPC1L1, a key protein in enterohepatic cholesterol absorption. J. Lipid Res. 50, 1653–62 (2009). 19325169 10.1194/jlr.M800669-JLR200 PMC2724045

[b28] Sparkes I. . Five Arabidopsis reticulon isoforms share endoplasmic reticulum location, topology, and membrane-shaping properties. Plant Cell 22, 1333–43 (2010). 20424177 10.1105/tpc.110.074385 PMC2879755

[b29] Jander G., Martin N. L. & Beckwith J. Two cysteines in each periplasmic domain of the membrane protein DsbB are required for its function in protein disulfide bond formation. EMBO J. 13, 5121–7 (1994). 7957076 10.1002/j.1460-2075.1994.tb06841.x PMC395459

[b30] Boyd D., Traxler B. & Beckwith J. Analysis of the topology of a membrane protein by using a minimum number of alkaline phosphatase fusions. J. Bacteriol. 175, 553–6 (1993). 8419303 10.1128/jb.175.2.553-556.1993 PMC196172

[b31] Duffy E. B. & Barquera B. Membrane topology mapping of the Na+-pumping NADH: quinone oxidoreductase from Vibrio cholerae by PhoA-green fluorescent protein fusion analysis. J. Bacteriol. 188, 8343–51 (2006). 17041063 10.1128/JB.01383-06 PMC1698230

[b32] Hanson B. R., Lowe B. A. & Neely M. N. Membrane topology and DNA-binding ability of the Streptococcal CpsA protein. J. Bacteriol. 193, 411–20 (2011). 21097630 10.1128/JB.01098-10 PMC3019820

[b33] Broome-Smith J. K., Tadayyon M. & Zhang Y. Beta-lactamase as a probe of membrane protein assembly and protein export. Mol. Microbiol. 4, 1637–44 (1990). 2077355 10.1111/j.1365-2958.1990.tb00540.x

[b34] Liu X. Y. & Matherly L. H. Analysis of membrane topology of the human reduced folate carrier protein by hemagglutinin epitope insertion and scanning glycosylation insertion mutagenesis. Biochim. Biophys. Acta 1564, 333–42 (2002). 12175915 10.1016/s0005-2736(02)00467-4

[b35] Wang H. . Membrane Topology of the Human Breast Cancer Resistance Protein (BCRP/ABCG2) Determined by Epitope Insertion and Immunofluorescence (dagger). Biochemistry doi: 10.1021/bi801644v (2008). PMC2649121 19063604

[b36] Hong M., Tanaka K., Pan Z., Ma J. & You G. Determination of the external loops and the cellular orientation of the N- and the C-termini of the human organic anion transporter hOAT1. Biochem. J. 401, 515–20 (2007). 17014423 10.1042/BJ20061171 PMC1820804

[b37] Chen J. G., Liu-Chen S. & Rudnick G. Determination of external loop topology in the serotonin transporter by site-directed chemical labeling. J. Biol. Chem. 273, 12675–81 (1998). 9575231 10.1074/jbc.273.20.12675

[b38] Tusnády G. E., Kalmár L. & Simon I. TOPDB: topology data bank of transmembrane proteins. Nucleic Acids Res. 36, D234–9 (2008). 17921502 10.1093/nar/gkm751 PMC2238857

[b39] Tusnády G. E., Dosztányi Z. & Simon I. PDB_TM: selection and membrane localization of transmembrane proteins in the protein data bank. Nucleic Acids Res. 33, D275–8 (2005). 15608195 10.1093/nar/gki002 PMC539956

[b40] Tusnády G. E., Dosztányi Z. & Simon I. Transmembrane proteins in the Protein Data Bank: identification and classification. Bioinformatics 20, 2964–72 (2004). 15180935 10.1093/bioinformatics/bth340

[b41] Weston S. . A membrane topology model for human interferon inducible transmembrane protein 1. PLoS One 9, e104341 (2014). 25105503 10.1371/journal.pone.0104341 PMC4126714

[b42] Roesli C., Mumprecht V., Neri D. & Detmar M. Identification of the surface-accessible, lineage-specific vascular proteome by two-dimensional peptide mapping. FASEB J. 22, 1933–1944 (2008). 18180333 10.1096/fj.07-100529

[b43] Niehage C. . The Cell Surface Proteome of Human Mesenchymal Stromal Cells. PLoS One 6, e20399 (2011). 21637820 10.1371/journal.pone.0020399 PMC3102717

[b44] Bausch-Fluck D., Hofmann A. & Wollscheid B. Cell surface capturing technologies for the surfaceome discovery of hepatocytes. Methods Mol. Biol. 909, 1–16 (2012). 22903705 10.1007/978-1-61779-959-4_1

[b45] Mirkowska P. . Leukemia surfaceome analysis reveals new disease-associated features. Blood 121, e149–59 (2013). 23649467 10.1182/blood-2012-11-468702

[b46] Karhemo P.-R. . An optimized isolation of biotinylated cell surface proteins reveals novel players in cancer metastasis. J. Proteomics 77, 87–100 (2012). 22813880 10.1016/j.jprot.2012.07.009 PMC3508169

[b47] Almahariq M. . Pharmacological inhibition and genetic knockdown of exchange protein directly activated by cAMP 1 reduce pancreatic cancer metastasis *in vivo*. Mol. Pharmacol. 87, 142–9 (2015). 25385424 10.1124/mol.114.095158 PMC4293446

[b48] Skvortsova T. E. . Cell-free and cell-bound circulating DNA in breast tumours: DNA quantification and analysis of tumour-related gene methylation. Br. J. Cancer 94, 1492–5 (2006). 16641902 10.1038/sj.bjc.6603117 PMC2361269

[b49] Semeraro F. . Extracellular histones promote thrombin generation through platelet-dependent mechanisms: involvement of platelet TLR2 and TLR4. Blood 118, 1952–61 (2011). 21673343 10.1182/blood-2011-03-343061 PMC3158722

[b50] Quillen M., Castello C., Krishan A. & Rubin R. W. Cell surface tubulin in leukemic cells: molecular structure, surface binding, turnover, cell cycle expression, and origin. J. Cell Biol. 101, 2345–54 (1985). 4066762 10.1083/jcb.101.6.2345 PMC2114015

[b51] Dudani A. K. & Ganz P. R. Endothelial cell surface actin serves as a binding site for plasminogen, tissue plasminogen activator and lipoprotein(a). Br. J. Haematol. 95, 168–78 (1996). 8857956 10.1046/j.1365-2141.1996.7482367.x

[b52] Hofmann A. . Surfaceome of classical Hodgkin and non-Hodgkin lymphoma. Proteomics. Clin. Appl. 9, 661–70 (2015). 26076441 10.1002/prca.201400146

[b53] Nury H. . Structural basis for lipid-mediated interactions between mitochondrial ADP/ATP carrier monomers. FEBS Lett. 579, 6031–6 (2005). 16226253 10.1016/j.febslet.2005.09.061

[b54] Deng D. . Crystal structure of the human glucose transporter GLUT1. Nature 510, 121–5 (2014). 24847886 10.1038/nature13306

[b55] Hegedűs T. . Inconsistencies in the red blood cell membrane proteome analysis: generation of a database for research and diagnostic applications. Database (Oxford). 2015, bav056 (2015). 26078478 10.1093/database/bav056 PMC4480073

[b56] Schatzmann H. J. & Rossi G. L. (Ca 2++ Mg 2+)-activated membrane ATPases in human red cells and their possible relations to cation transport. Biochim. Biophys. Acta 241, 379–92 (1971). 4258479 10.1016/0005-2736(71)90037-x

[b57] Wolf H. U. Studies on a Ca 2+-dependent ATPase of human erythrocyte membranes. Effects of Ca 2+ and H+. Biochim. Biophys. Acta 266, 361–75 (1972). 4261006 10.1016/0005-2736(72)90094-6

[b58] Lowry O. H., Rosebrough N. J., Farr A. L. & Randall R. J. Protein measurement with the Folin phenol reagent. J. Biol. Chem. 193, 265–75 (1951). 14907713

[b59] Guan S., Price J. C., Prusiner S. B., Ghaemmaghami S. & Burlingame A. L. A data processing pipeline for mammalian proteome dynamics studies using stable isotope metabolic labeling. Mol. Cell. Proteomics 10, M111.010728 (2011). 10.1074/mcp.M111.010728 PMC3237081 21937731

