# Supplementary material for: Identification of Extracellular Segments by Mass Spectrometry Improves Topology Prediction of Transmembrane Proteins
Source: Sci Rep. 2017 Feb 13;7:42610. doi: 10.1038/srep42610 (PMC5304180; doi:10.1038/srep42610)

Supplementary Information for

IDENTIFICATION OF EXTRACELLULAR SEGMENTS BY MASS SPECTROMETRY IMPROVES TOPOLOGY PREDICTION OF TRANSMEMBRANE PROTEINS

Tamás Langó, Gergely Róna, Éva Hunyadi-Gulyás, Lilla Turiák, Julia Varga, László Dobson, György Várady, László Drahos, Beáta G. Vértessy, Katalin F. Medzihradszky, Gergely Szakács and Gábor E. Tusnády

Supplementary Methods:

Verification of cell surface labeling by microscopy

Successful labeling was verified by confocal microscopy. Labeled cells were blocked with 6% BSA (to prevent non-specific binding of avidins) in PBS for 10 minutes at 4°C; samples were incubated with TexasRed-conjugated avidin (Vector Laboratories, 1:500 for 1 hour, at 4°C). CHO cells were counterstained with Hoechst 33342 DNA dye (0.1 µg/ml in PBS) for 2 minutes. Images were acquired with a Zeiss LSCM 710 microscope using a 63x NA=1.4 Plan Apo objective (Supplementary Figures1 and 2).

Optimization of surface biotinylation with flow cytometry analysis

The concentration of the biotinylation agent was optimized by flow cytometry to achieve maximum efficiency without toxicity. Equal numbers of cells were incubated with increasing concentrations of sulfo-NHS-SS-biotin (raging between 12.5 µM and 2.5 mM). After quenching with 100 mM TRIS.HCl pH=7.4, 150 mM NaCl for 10 minutes, samples were blocked with 6% BSA in PBS for 10 minutes at 4°C. Biotinylated side chains were visualized by incubating the samples for 1.5 hours at 4°C with Alexa-488 conjugated streptavidin in blocking buffer (1:500, Sigma). After washing with PBS, mean fluorescence intensity values, reflecting cell surface

biotinylation efficiency, were measured. Viability and integrity of the cells was monitored with the lack of propidium iodide staining during labeling (Supplementary Figure 3).

Dot-blot analysis

The binding capacity of the neutravidin agarose column was tested with dot-blots. The biotin content of each membrane preparation was monitored before and after affinity isolation and purification. These samples were diluted 100-fold (the samples used for calibration were prepared as: 1%, 2%, 10%, 25%, 50%, 75%, 90%, 100% of the diluted samples before avidin column purification) and spotted onto PVDF membrane using a Bio-Dot microfiltration apparatus (Bio-Rad, Hercules, CA, USA). Membranes were blocked in blocking buffer (25 mM TRIS.HCl, pH=7.4, 2.7 mM KCl, 137 mM NaCl, 0.05% Tween-20) containing 6% BSA for 45 minutes at room temperature. Membranes were further incubated with HRP-conjugated avidin (1:50000, Sigma) for 45 minutes at room temperature in blocking buffer. The biotinylated peptides were visualized using the enhanced chemiluminescence reagent (GE Healthcare, Buckinghamshire, UK) and images were captured by a BioRad ChemiDoc™ MP Imaging system (Supplementary Figure 4).

Supplementary Table 1: Structure of the applied lysine modifications.

Supplementary Table 2: Information about raw files and the lists of the identified peptides that were modified on lysines.

Supplementary Table 3: List of transmembrane proteins filtered to 95% sequence identity and their labeled positions.

Supplementary Figure 1: CHO cell line tested for cell surface protein labeling with TexasRed conjugated avidin (Panel A: Hoechst (DNA marker), Panel B: DIC, Panel C: Labeled

extracellular surface (TexasRed), Panel D: Merged). Labeled membrane preparations were tested with Texas Red conjugated avidin (Panel E: DIC, Panel F: Labeled extracellular surface (TexasRed)).

Supplementary Figure 2: Unlabeled CHO cells were treated with Texas Red conjugated avidin and were analyzed with confocal microscopy (Panel A: Hoechst (DNA marker), Panel B: DIC, Panel C: Labeled extracellular surface (TexasRed), Panel D: Merge).

Supplementary Figure 3: A) Percentage of „dead” (PI positive) cells with or without labelling (*ns=not significant, by non-parametric Mann-Whitney test). B) Saturation curve of cell surface labeling measured by flow cytometry. Arrows indicate samples from which membrane fractions were prepared for the dot-blot assay.

Supplementary Figure 4: Dot-blot assay to measure the biotin content of samples

Line A shows labeling of a sequentially diluted sample of known biotin content (see Experimental procedures) to validate the flow cytometry results (line B) and to test the neutravidin resin's binding capacity (line C). B1: Negative control (non-biotinylated sample). B2 to B6: representative labeled membrane preparations (1-5) from the saturation curve of the flow cytometry measurement (Supplementary Figure 3B), B7-B8: Positive controls (biotinylated samples). Line C: samples before affinity isolation and samples that passed through the neutravidin columns. Fractions of MS Grade trypsin (C1-C2), chymotrypsin (C3-C4) and thermolysin (C5-C6) digestion from K562 cell line. C7-C8: unlabeled membrane preparations (negative control).

Supplementary Figure 5: Sequence logo of the environment of labeled lysines. Lysines expected to be detected by mass spectrometry were determined as follows: located in the

extracellular region of the transmembrane proteins, semi-tryptic peptide with maximum four modifications and m/z between 380-1600 and charge between 2-4. Then, the ±10 amino acid environment of their position was cut out (excluding the lysine residue in question). By comparing the environment of expected and found lysines, we created a sequence logo for the preceding and succeeding sequences. We analyzed the sequences encompassing the lysines from two aspects: i. whether we expected the particular lysine to be found and ii. we virtually found it. To avoid bias, all sequences were filtered to 40% identity by CD-HIT (59). The sequence logos were created with Seq2Logo (60).

Supplementary Figure 6: Frequency distribution of the lengths of extracellular segments containing covalently modified lysines relative to the total number of extracellular loops containing lysines (blue). Captured lysine positions were randomized in extracellular loops according to the hit rate of the method, then extracellular segments containing randomly selected lysines were divided by the number of extracellular loops containing lysines. The randomization was repeated 1000 times and distribution of segment lengths was calculated. Average and standard deviations are plotted (red). To avoid bias, all sequences were filtered to 40% identity by CD-HIT (59).

Supplementary Figure 7: Predictions were sorted according to their reliability values, and then a subset with the 1, 2, 3 … 333 highest realibilites were chosen, represented as coverage from 0 to 100% on the x-axis of the plot. For each subset, the lowest reliability was plotted against the coverage of the subsetThe colors of the curves are coded according to the ratio of randomly selected extracellular lysine (blue, green, orange, red and black for 100, 75, 50, 25 and 0%, respectively). Averages are shown with line, standard deviations are shaded.

Supplementary Figure 8: Homologous structure for ADT2_HUMAN (the bovine mitochondrial ADP-ATP carrier, PDB: 2C3E), colors are based on topology (blue: extracellular; yellow: membrane; red: cytosolic). Captured lysines are marked with purple, in parenthesis their position

in the structure and the number of corresponding peptide hits from MS are shown.

Supplementary Figure 1

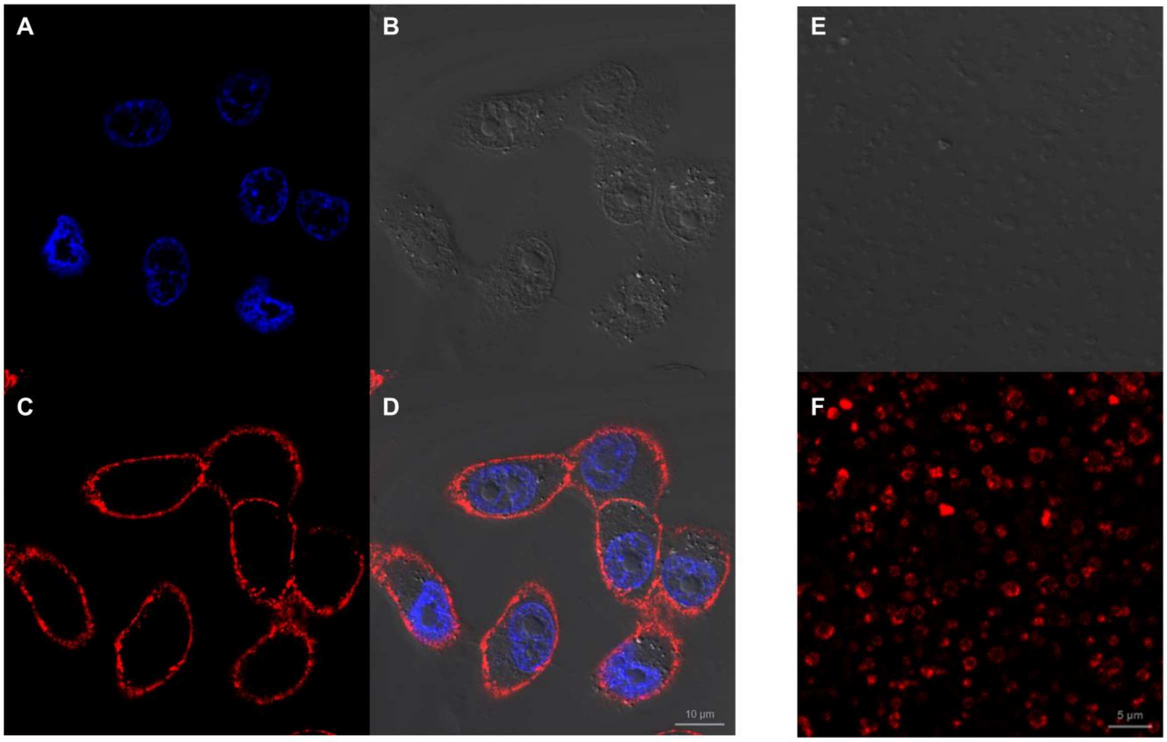

Supplementary Figure 2

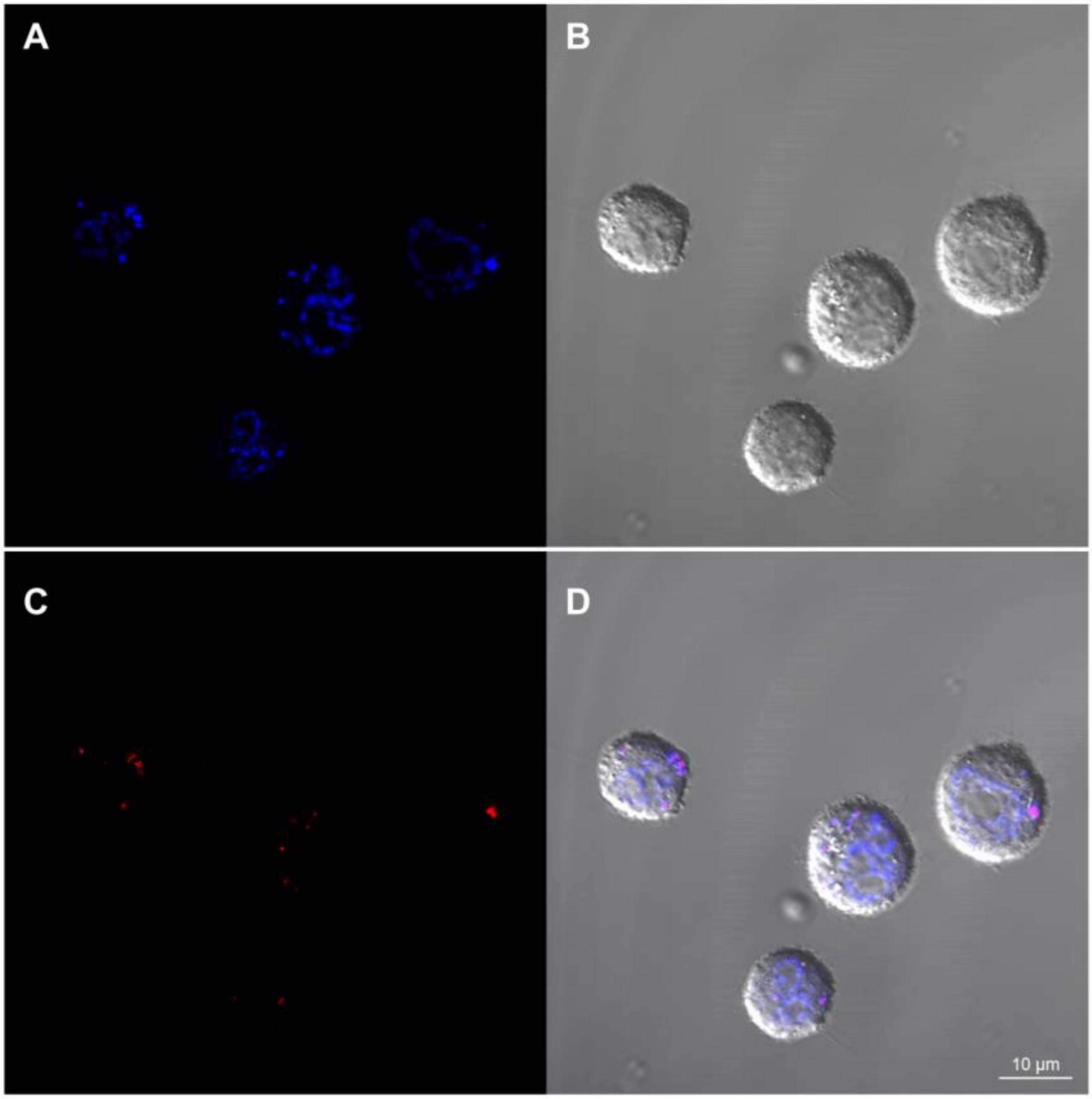

Supplementary Figure 3

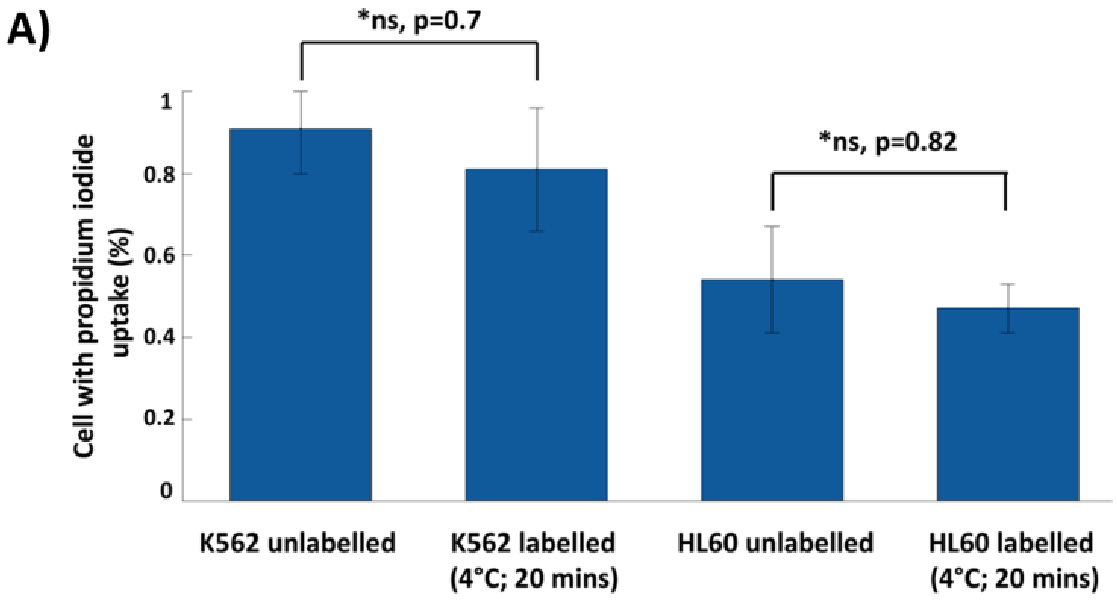

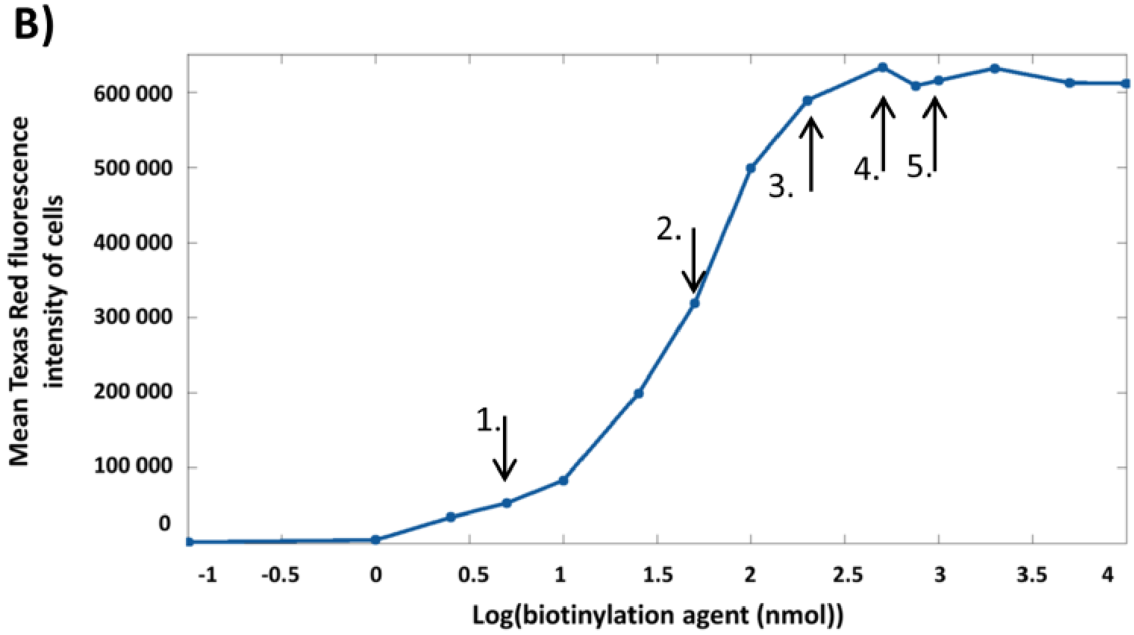

Supplementary Figure 4

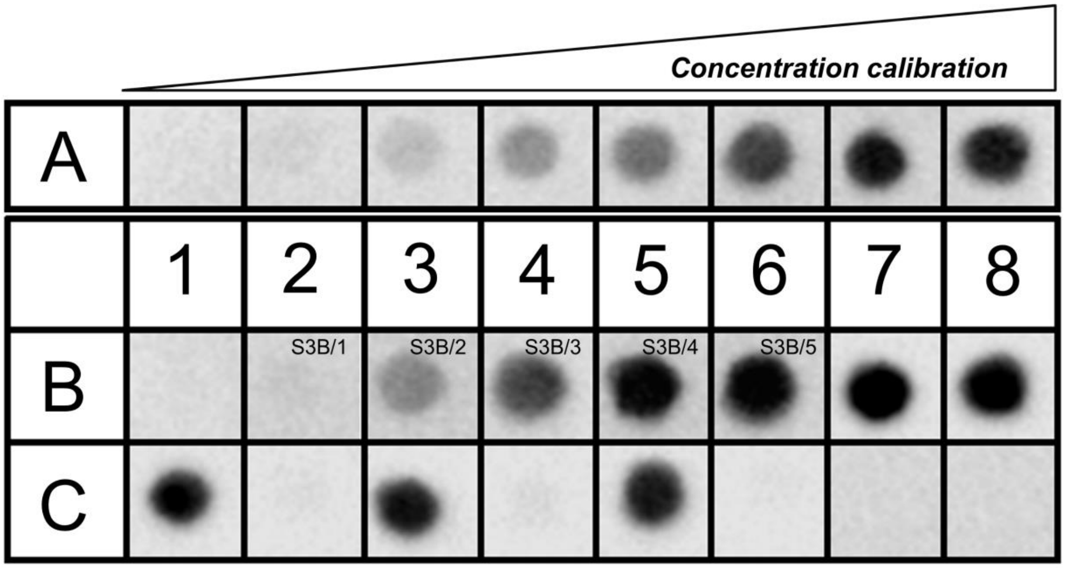

Supplementary Figure 5

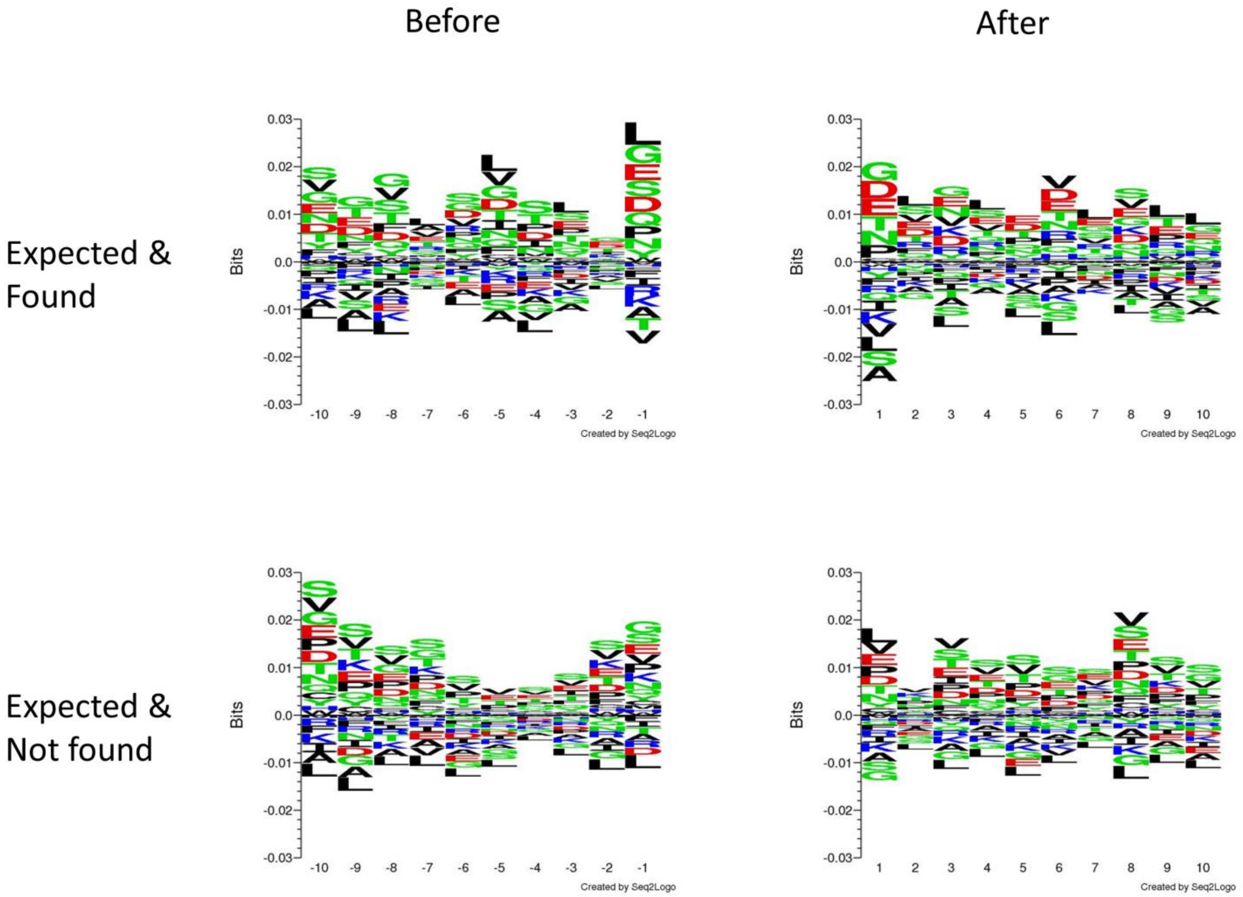

Supplementary Figure 6

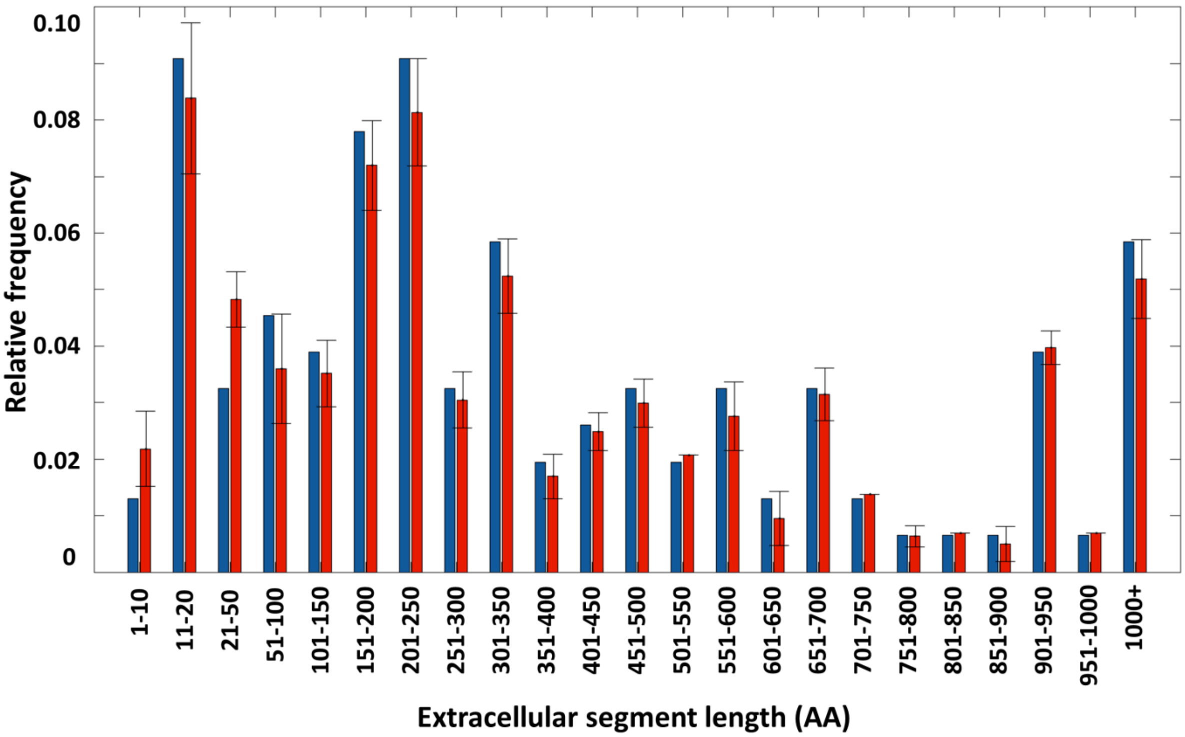

Supplementary Figure 7

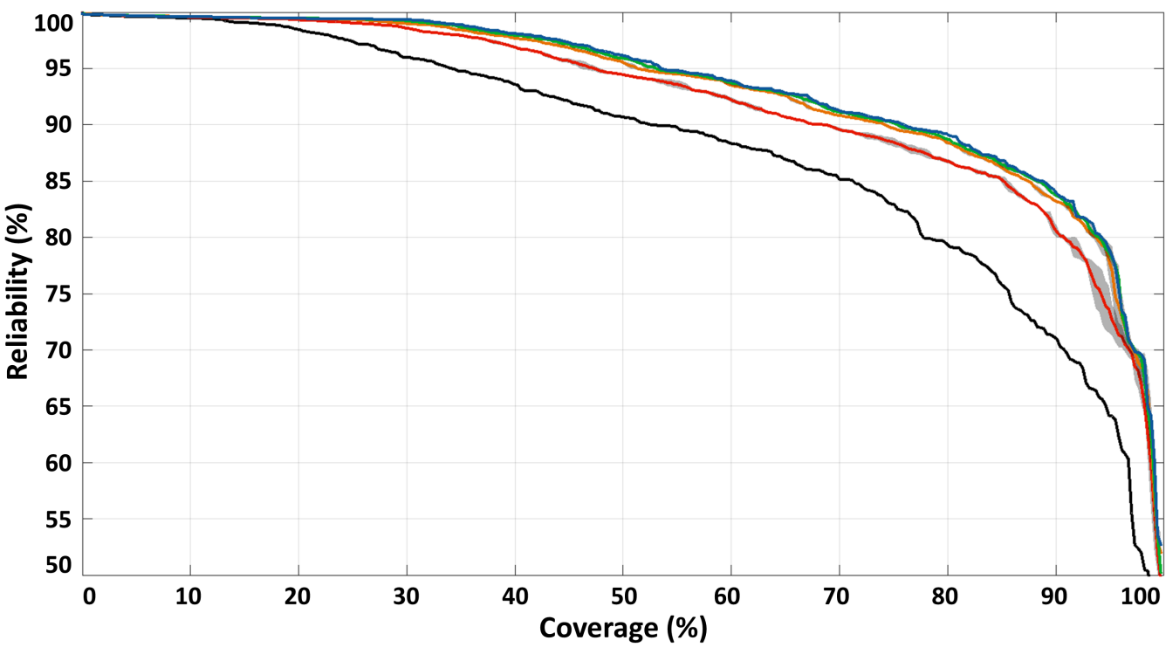

Supplementary Figure 8

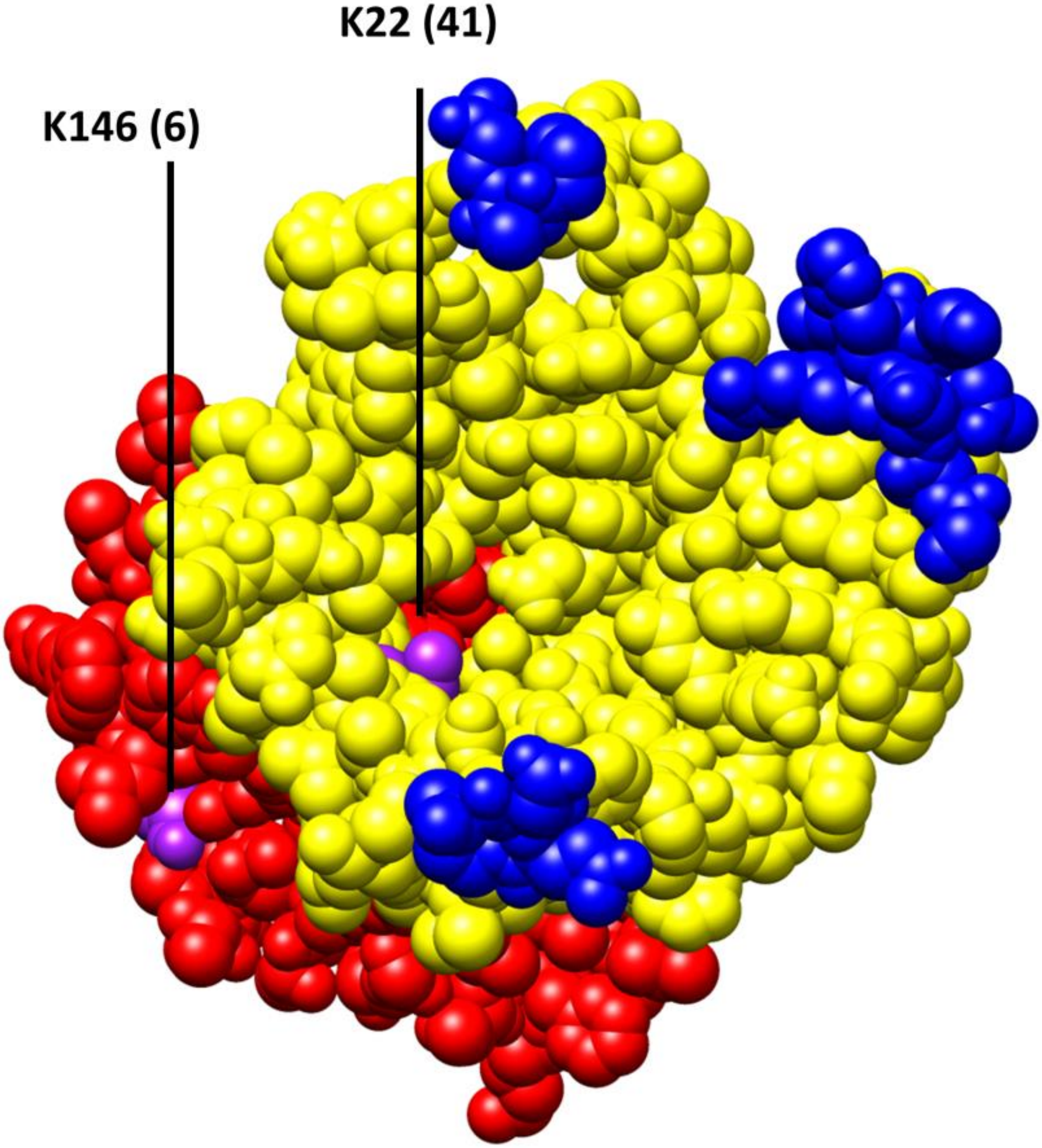

Supplement: Supplementary Data [file srep42610-s1.pdf]
